# Reproduction of Bone

**Published:** 1870-09

**Authors:** 


### Selections.

---

Reproduction of Bone.—At a meeting of the Medical Society of the County of New York, (*Medical Record*), Dr. Wm. R. Whitehead read a paper on this subject. Assuming that the conditions which regulate bone growth, could we fully discover them, would be found as constant in their action as those which govern the phenomena of inorganic nature, the speaker proceeded to give his views of the true method of investigation to be pursued, and to review the history of opinion upon the subject of osteogenesis, touching also upon the general history of experimental research in medicine and its cognate sciences. It was important to seek for the minor laws that modify the working of the more general ones, producing perturbations which, whether seen in the growth of a cell, in the formation of a crystal, or in the motions of a planet, will prove, when these laws are discovered, to be not fortuitous, but regular. And to aid in these researches it was important to bring the results of a broad generalization to bear upon each limited department of study.

To Duhamel, in the last century, when France was the theater of the highest intellectual activity, was due the discovery of the property of the periosteum to reproduce bone. The theory suggested by his studies in vegetable physiology he established by experiment. It was opposed by his cotemporaries, and rejected by Bichat and Scarpa, who for a time held almost undisputed sway over medical opinion. Later, Dupuytren admitted the action of the periosteum in forming the "provisional callus;" but it was only about a century after Duhamel's discovery that it was forced into general recognition by the efforts of many investigators, prominent among whom was Flourens. At present Sedillot is the leading opponent of the doctrine that the detached periosteum has the power to reproduce bone, maintaining that the only way of insuring regeneration, in cases of necrosis, is to scoop out (*evider*) the bone in such a manner as to leave its outer layer still adhering to the periosteum.

He has been ably refuted, and the efficacy of subperiosteal resection fully established by Holmes and many others. Among the surgeons in this city whose experience upon this point would seem to be conclusive, may be mentioned Dr. James R. Wood.

But the repair of bone is not left dependent upon the periosteum alone. The medullary tissue has a similar power, which, as Goujon has conclusively shown, it may exhibit after transplantation into vascular parts, especially in early life, when it contains an abundance of calcareous salts. But it is far less likely to produce bone than is the periosteum, under similar conditions, and the bone produced is soon absorbed, while that originating from periosteum is apt to be permanent. As to the so-called medullary membrane, or endosteum, its functions, whether of reproduction or absorption, can not be regarded as settled; and indeed its very existence is in dispute.

Although the medullary tissue stands next to the periosteum in osteogenetic power, yet it shares this with nearly all cellular and fibrous tissues, and ever muscular tissue may become ossified under favoring conditions. These conditions would seem to be chiefly long continued or repeated irritation, in connection with "a perverted assimilation of the calcareous salts of the blood." The presence of some of the proper osseous tissues in a state of irritation is also regarded by Ollier as conducive to osssification of the neighboring parts.

The effort to obtain reproduction of bone by transplantation of periosteal flaps (as distinguished from subperiosteal resection), so successful in many of the experiments upon animals, has not commonly met with the same success in man. Still, its satisfactory results, under good conditions, are sufficiently numerous to warrant its recognition as an important surgical procedure, likely in the future to receive a wider application. At present, perhaps the best illustrations of its value are to be seen in Langenbeck's operation for closure of cleft of the hard palate, known as muco-periosteal aranoplasty. The speaker had—in two cases of this operation which he had published—convinced himself that the cleft in the palate might thus be closed by a growth of new bone.

In conclusion, Dr. Whitehead related three experiments of subperiosteal resection which he had made upon rabbits, and exhibited the specimens, showing their results.

Dr. J. C. Nott said that some thirty years ago Dr. Toner, now of California, then a young man commencing practice, had published several cases of onychia, in which he had removed the phalanx, leaving the periosteum, and obtained a reproduction of the bone. Impressed by these cases, the speaker had himself followed this practice ever since, and with success. He had heard that old Dr. Dudley was accustomed to treat onychia in the same manner. In a case of necrosis of the upper jaw he had, in removing the bone, left its periosteum in situ, and the bone was regenerated.

Dr. Sayre had from his earliest practice removed phalanges in the manner above described, which he had been taught by Dr. Dudley. It was very important, in such cases, to dress the finger, during the process of healing, so as to preserve its normal shape, otherwise the nail would become hooked over, or some other deformity result. One of the speaker's sons had smashed his finger throughout its whole length, so that at first amputation seemed unavoidable. But by careful preservation of all the periosteum almost complete reproduction of all the phalanges was secured, and the finger was now serviceable, though somewhat shorter than its fellows. The doctor had resected subperiosteally four and one-half inches of the femur of a young man. The bone was completely reproduced, the limb equal in length to the other; and the lad had last winter won a prize in a skating match.

Dr. Chadsey related a case of necrosis of the entire tibia, which was removed as a sequestrum, new bone having formed around it firm and serviceable, though at first large and misshapen. It gradually became reduced to about the natural size. A patient of his had been kicked by a horse above the eyebrow, severely comminuting the outer table. In removing the pieces of bone, the periosteum was left attached to the skin; and the lost bone was restored with no depression or deformity.

Dr. Bibbins referred to two cases of necrosis of the lower jaw, from gangrenous stomatitis in children which had come under his care at the Nursery Hospital, Randall's Island, in 1850. In one of them he had resected the bone from the canine tooth to the angle of the same side; in the other, from the canine tooth to the angle of the opposite side. The periosteum was carefully left in both cases, and in both the part removed was reproduced, though, of course, without teeth or alveolar process.

